# Effects of sea ice on growth rates of an endangered population of gray whales

**DOI:** 10.1038/s41598-020-58435-3

**Published:** 2020-01-31

**Authors:** G. Gailey, O. Sychenko, O. Tyurneva, Y. Yakovlev, V. Vertyankin, P. van der Wolf, K. Drozdov, I. Zhmaev

**Affiliations:** 1 Cetacean EcoSystem Research, Olympia, WA USA; 2 National Science Center of Marine Biology, Far East Branch, Russian Academy of Sciences, Vladivostok, Russia; 3 Kronotsky State Biosphere Reserve, Elizovo, Russia; 4 Consultant to Sakhalin Energy Investment Company, Yuzhno-Sakhalinsk, Russia; 5 LGL Eco, Vladivostok, Russia

**Keywords:** Conservation biology, Population dynamics

## Abstract

The western gray whale population is endangered with approximately 175 individuals and 33 known reproductive females. Photo-identification studies were conducted from 2002–2017 during the gray whale foraging season off northeastern Sakhalin Island, Russia. Despite abundant prey resources, significant variation in whales’ body condition, inter-birth intervals and calf survival have been documented with limited understanding of factors that account for the observed variability. We examine sea ice concentrations at their known foraging grounds to define the maximum duration of a “foraging season”. We explore the relationship between foraging season length during a female’s pregnancy and post-weaning calf survival and reproduction. Approximately 77% of the variation in calf survival, which ranged annually from 10–80%, was associated with the duration of the feeding season while the mother was pregnant. Poor body conditions and prolonged inter-birth intervals of western gray whales have also been documented to coincide with shorter duration in feeding seasons found in this study. These results imply that shorter foraging seasons are associated with reduced energy intake by physically limiting the number of days gray whales can forage, and thus sea ice conditions may be one limiting factor affecting growth rates of this endangered population of baleen whales.

## Introduction

There are two genetically distinct populations of gray whales (*Eschrichtius robustus*) that reside only in the North Pacific Ocean^1–5^. On the eastern side of the North Pacific Ocean, gray whale recovery from commercial whaling has been a conservation success story with population sizes reaching pre-whaling numbers^6^. However, gray whales on the western side of the North Pacific remain a small remnant of their historical population estimates^7^ and are currently listed as endangered by IUCN with a population size of 175 individuals, with only 33 known reproductive females^8,9^. In the past two decades, the western population has increased in size at an average rate of 2–4% per annum with recruitment almost exclusively from calf production with little to no immigration^8^. Despite the overall positive growth rate, several demographic parameters suggest that the western population are not realizing their maximum productivity levels with low post-weaning calf survivability and prolonged inter-birth intervals for reproductive females^1,10,11^. In addition, body conditions of western gray whales have notably varied over time with significantly worse conditions prevalent in some years compared to others^12^. Although anthropogenic activity has been hypothesized to limit pregnancy and calf survival rates^8^, other natural factors such as prey availability and/or access to their foraging habitat have also been suggested to explain the variability in population growth rates and reproductive success^12^.

Gray whales are capital breeders that forage at high latitudes during summer months and breed/calve at lower latitudes during winter months with little-to-no feeding occurring outside of their foraging grounds. Upon leaving their feeding habitats, gray whales depend on the energy obtained from their foraging period to sustain them during their north/south bound migrations as well as time spent on the breeding grounds^13^. Energetic requirements are particularly demanding for pregnant females with a 13 month gestation period and calves being weaned in the subsequent feeding season^14,15^. Other baleen whales have been noted to lose an average of 25% of their initial body volume during a three month breeding season, which highlights the energetic demands on reproductive females^16^. Gray whale energetic studies found that females would wean calves at ~37% energetic loss and prolong subsequent pregnancies at 30–35% energetic loss due to lactation and calf rearing^17^. In addition to the energetic requirements of reproduction, western gray whales may have higher energetic requirements compared to their eastern counterparts if they spend more time on the breeding grounds, where energetic requirements are high, along the coast of Asia, which could further contribute to their slow recovery^15^.

The primary foraging grounds of the western population of gray whales is off northeastern Sakhalin Island, Russia. Off Sakhalin, gray whales utilize a nearshore and offshore feeding area which are exceptionally high in benthic prey resources with little-to-no significant inter-annual change that could constrain reproductive success and overall growth rates^18^. However, unlike the eastern gray whale foraging grounds, the western gray whale foraging habitat is drastically smaller geographically, spatially static and more susceptible to environmental perturbations. In fact, the western gray whale nearshore and offshore feeding habitats are approximately 600 and 700 km^2^, respectively, with foraging frequently observed in the nearshore feeding area, while the offshore utilization has been more variable.

Gray whales have been suggested to be “ecosystem sentinels” due to their documented sensitivities and responses to environmental changes^19–22^. Annual sea ice conditions likely play an important role in gray whale prey production and access to prey. The influence of sea ice has been noted to affect a number of polar marine mammal species^23–30^. Sea ice extent in the Bering Sea has been suggested to limit calf production in eastern gray whales with a significant decrease in number of calves during shorter feeding seasons when pregnant females were foraging. Approximately 70% of the inter-annual variation in the estimated number of northbound calves of eastern gray whales were explained by the average sea ice condition in the Bering Sea in the year prior to birth^19,31^. This suggests that existing pregnancies were impacted during poor foraging conditions compared to suppression of ovulation as suggested by Rice and Wolman^32^. On the foraging grounds of the western gray whales, ice-free conditions typically occur from early-mid May to mid-December, although substantial annual variation in sea ice concentration and extent exists in the Sea of Okhotsk^33^. In this study, we examine the variability of sea ice conditions on the western gray whales foraging grounds and explore potential associations with calf recruitment and, thus growth rates of this population.

## Results

### Calf production and recruitment

**Figure 1:**
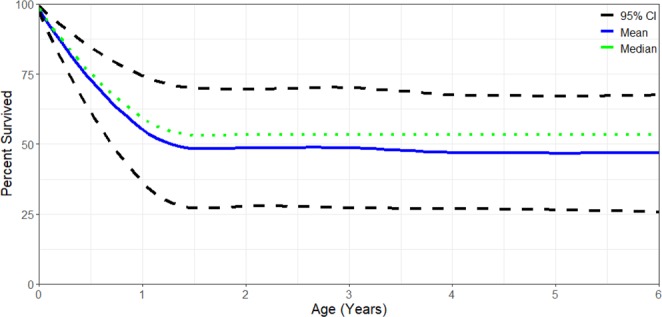
Western gray whale calf survivorship curve from 2002–2013.

A total of 82 calves were recorded on the foraging grounds from 2002–2013 with a mean of 7.5 ± 3.45 SD calves ranging from 3 to 15 individuals per year. Given a pool of 33 known reproductive females, a median of 54% of the calves consistently returned to the Sakhalin feeding area in the subsequent 4 years and thus contributed to the population size of western gray whales. The vast majority of the initial decline in calf survivability occurred during the first year of life (Fig. 1). Annual variation in calf survival ranged from 10–80% of the number of calves observed in their first year of life.

### Sea ice concentration and feeding days

**Figure 2:**
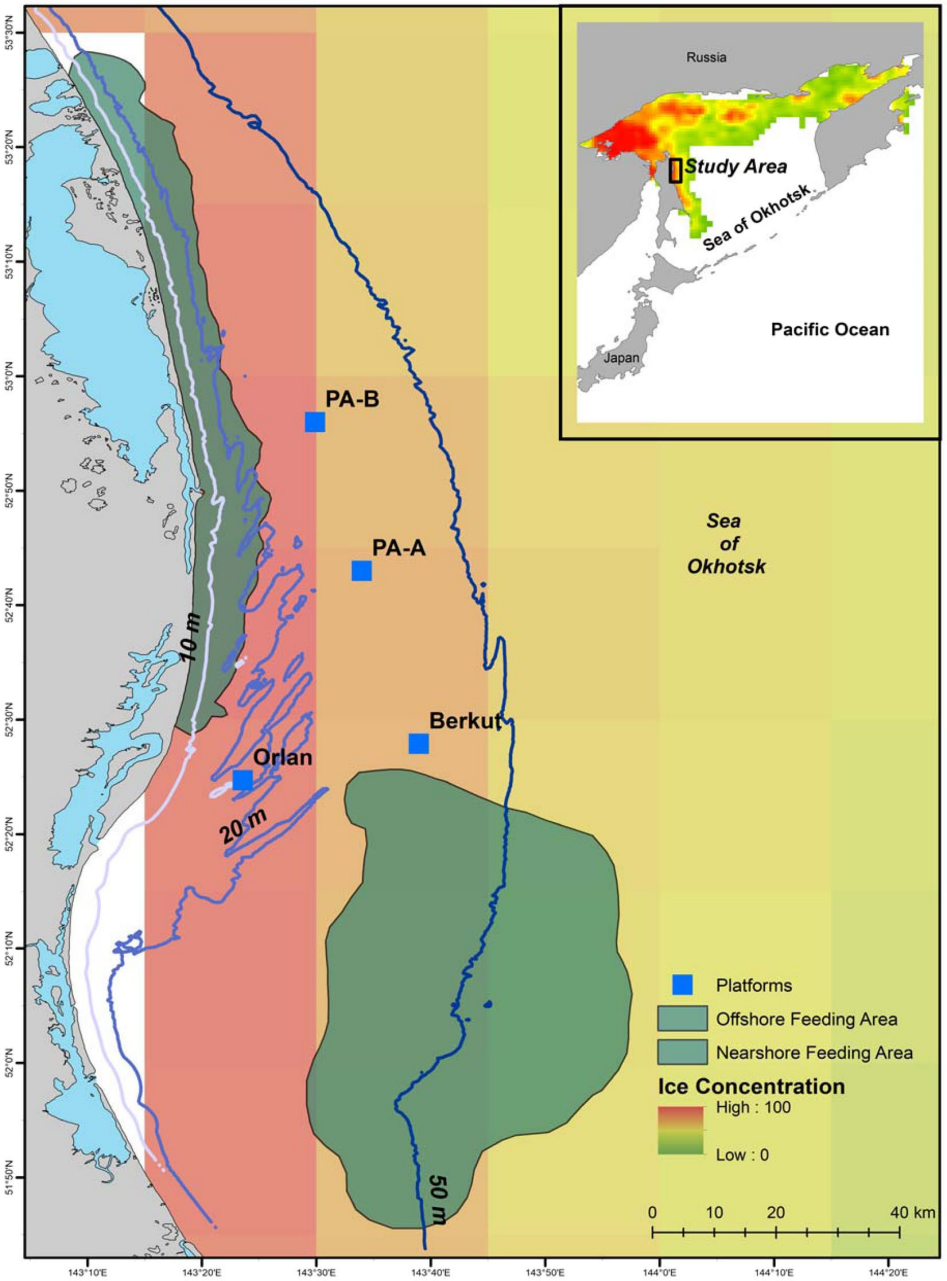
Sea ice concentration (%) off northeastern Sakhalin Island, Russia on 25 May 2010 relative to the historically known western gray whale nearshore and offshore foraging habitats. Oil platforms that are in close proximity to the feeding grounds are identified as blue squares. Grid cells with missing values are designated as transparent.

**Figure 3:**
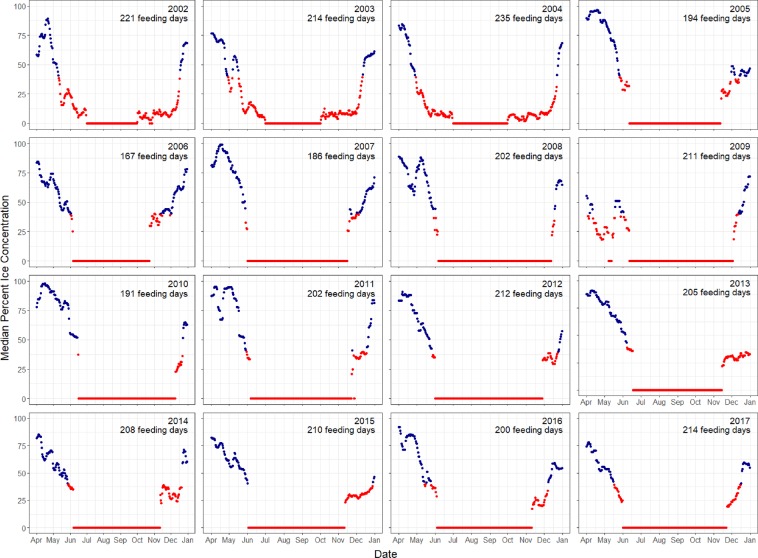
Daily median sea ice concentration from 2002–2017 in the nearshore feeding area. Red points indicate gray whale foraging opportunities while blue points (>40% sea ice concentration) indicate non-foraging days.

Daily median ice concentration values were extracted for grid cells overlapping the western gray whale nearshore feeding habitat. Figure 2 illustrates a representative ice concentration grid recorded on 25 May 2010. The median daily ice concentration values from 2002–2017 in the western gray whale foraging grounds varied annually with typical “ice free” (e.g. ≤40%) periods occurring around early-mid May with the onset of ice formation occurring around early-mid December (Fig. 3). The mean number of potential gray whale “feeding” days off northeastern Sakhalin was 204 ± 15.5 SD days ranging from 167 to 235 days.

### Sea ice and calf survival

**Figure 4:**
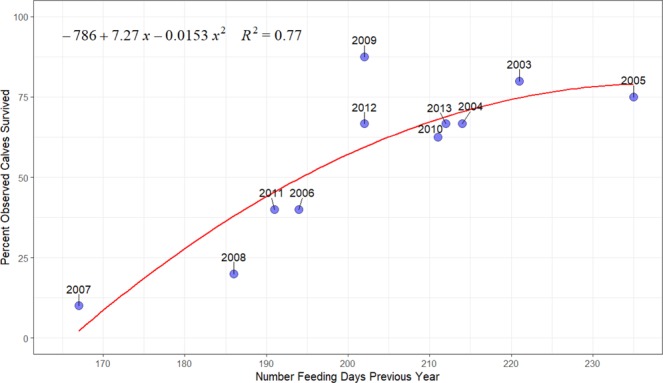
Number of feeding days during pregnancy, based on ice concentrations, compared to the percentage of calves that survived their first year post-weaning. Year represents the first time calves were observed (e.g. one year after the feeding season of their mother’s pregnancy). The year 2002 was excluded due to no calves being observed that year.

At the end of a foraging season, a fasting and pregnant female relies solely on stored energy she acquired during the foraging season when she was pregnant (plus any additional stores she may already have) to successfully bring a calf back to the feeding grounds the following year. Examining post-weaning calf survival as a function of sea ice coverage during pregnancy provides a potential link between possible poor energy intake during pregnancy due to ice, reduced female condition and lower calf survival even after weaning has occurred. Calf survival was significantly correlated (p = 0.007, R^2^ = 0.77) with the number of feeding days that their mothers experienced during the previous foraging season when they were pregnant (Fig. 4). No significant association was found using the number of foraging days during the year the individual was observed as a calf (p = 0.412, R^2^ = 0.22) or two years prior to the observed calf arriving on the feeding grounds (p = 0.490, R^2^ = 0.19). Sensitivity analyses indicated that significant associations between calf survival and the number of feeding days during the mother’s pregnancy were found with median ice concentration values for feeding days being defined as 10–40% concentration with the strongest relationship occurring with 40% median concentration criteria. The definition of feeding days using higher median ice concentrations (e.g. >40%) showed no significant association with calf survivability, which could suggest that gray whale feeding may have been inhibited at these higher median ice concentration levels.

## Discussion

Annual sea ice conditions were found to account for 77% of the annual variability in calf survival in western gray whales. These results are comparable to the effects of sea ice conditions found for eastern gray whales. Perryman *et al*.^19,31^ found that sea ice extent in the Bering Sea accounted for 70% of the variation in calf production, as estimated during their northbound migration. The total number of calves produced annually (calf production) was linearly correlated to sea ice conditions during the previous year. Perryman *et al*.^19,31^ examined the number of calves that had survived to reach central California. Our calf counts represent the number of calves that survived to reach the foraging grounds, after potential additional mortality, such as predation, may have occurred. In addition, the small number of females that may be pregnant in any given year means individual stochasticity plays an important role in calf counts. The total number of calves off Sakhalin was not associated with the number of feeding days in the previous foraging season. In fact, the highest number of observed calves (15) was recorded in 2011, but only five of those calves were observed to continuously return to Sakhalin foraging grounds in subsequent years. The number of calves in this study was not likely representative of true calf production due to variable photo-identification research effort on the foraging grounds^34^ and mortality events during their first 6–7 months of life. It is possible that calf production was impacted by shorter feeding seasons prior to individuals reaching the feeding grounds while further decreased survival of the calves continued even after calves reached the feeding grounds with their mothers. In addition to providing less nutrients to calves during the migration, struggling lactating females that arrive with a calf could potentially wean calves earlier due to insufficient energy reserves to further support the calf. Early weaning could result in decreased calf growth and survival and, thus, could impact overall population growth.

This study is the first to examine the potential duration of the gray whale foraging season off Sakhalin, as determined by sea ice conditions. The observed ice conditions on their feeding grounds may have limited access to prey resources for prolonged periods of time in some years. In particular, pregnant females feeding in 2005–2007 and 2010 had less than a 200 day foraging season. Gray whale energetic models assume a pregnant female would need approximately 200 foraging days to sustain them and their calf^15,17^. Therefore, these shorter than average foraging seasons could explain lower than average survival of calves in 2006–2008 and 2011. Pregnant females who experience limited access to the foraging habitat early in the season could potentially “compensate” by either foraging more efficiently or staying later in the foraging season. However, when ice conditions early in the feeding season are prolonged and ice formation builds up earlier in the same feeding season off northeastern Sakhalin, this limits compensation opportunities. Females could also risk reproductive success by extending their foraging period as they could give birth outside of the breeding grounds which could increase predation of their calf by killer whales (*Orcinus orca*) as well as place thermoregulatory stress on the newborn calf. In recent years, eastern gray whale calves were recorded to have been born north of their breeding grounds which has been suggested as a result of delayed southbound migration due to increased competition for food resources or limited early access to prey due to sea ice conditions^35^. The results of this study suggest gray whales will compensate to some degree as the shorter feeding seasons were a result of both prolonged ice conditions early in the season coupled with early arrival of sea ice in the same year. In other years, such as 2013, sea ice was extended early in the feeding season to mid-June, but a rather mild onset of ice arrival in December could have allowed females to extend their foraging opportunities and thus increased the probability of survival of their calf. This study further suggest that gray whale compensation during prolonged ice conditions early in the feeding season is more likely a result of staying on the foraging grounds longer rather than by the possibility of individuals feeding more efficiently. While this study only considered calf survival if they returned to the Sakhalin foraging grounds, the unobserved calves could have survived and emigrated. In fact, limited data collected off Kamchatka, Russia in 2012–2013 identified three calves from 2011 that have never been observed to have returned to Sakhalin in subsequent years^36,37^. Although these calves may have survived, they ultimately did not contribute to growth rates of the western gray whale population.

Bradford *et al*.^12^ found significant annual variation in body condition in western gray whales which they hypothesized to be related to environmental limitation(s) or density-dependent regulation. Body conditions of western gray whales were significantly better in 2004 and worse in 1999, 2006 and 2007^12^. Poorer body conditions in 2006–2007 coincide with shorter foraging seasons in the previous year as estimated in this study, suggesting females return from fasting in a poorer nutritive state if the foraging season prior was shorter in duration. Conversely, foraging opportunities in 2003–2004 represented longer foraging seasons, which could explain the significantly improved body conditions observed in 2004. Although this study was limited to photo-identification data obtained from 2002–2017, sea ice concentrations in 1998 (219 feeding days) do not appear to account for poor body conditions observed in 1999 (213 feeding days) by Bradford *et al*.^12^. Eastern gray whales experienced an unusually high mortality event in 1999/2000 which was hypothesized to be related to prey availability or alternatively climate effects in the North Pacific Ocean^38,39^, although there are no prey data from that time period. Of the three calves identified by Bradford *et al*.^12^ in 1999, none were recorded to have returned to the Sakhalin foraging grounds (D. Weller and A. Burdin, personal communication). Therefore, sea ice conditions alone do not appear to account for the anomalous calf survival or the whales’ body condition in 1999.

Western gray whale population modelling efforts have further documented an exceptionally low pregnancy rate in 2008 as well as low calf survival/annual return of calves in 2007^8^. Cooke *et al*.^8^ hypothesized that low recruitment could be related to anthropogenic activity (pile driving) that was conducted in 2008. This hypothesis seems unjustified for calf survival as pile driving activity in 2008 was localized and short in duration with relatively low sound exposure levels compared to pile driving activity in the subsequent year, which was more extensive. In addition, other known activities, such as seismic surveys, would have likely had more of an impact on calf survival due to the larger spatial extent, longer duration and higher sound exposure levels^40–47^. Restricted access to their feeding area due to ice conditions in 2005–2007 would be a more plausible explanation to the unusually low calf survival and pregnancy rates in 2007–2008 found in population models.

In addition to calf survival and body condition, western gray whales have prolonged and highly variable inter-birth intervals^1,10,11^. Based on photo-identification data from 2002–2017, a total of 24 two-year and 9 three-year inter-birth intervals have been recorded to date^34^. Seven of the nine (77%) of the longer three-year birth intervals occurred to mothers known to have given birth after a shorter-than-usual feeding season during their pregnancy. This could suggest that a pregnant female’s reproductive success may not only decrease due to a lower probability of calf survival, but shorter foraging seasons may also result in a delay of successive pregnancies due to a compromised body condition and the need to replenish reserves.

Impacts from anthropogenic activity related to oil and gas development near the western gray whale feeding grounds could have also contributed to the observed calf survival. Foraging seasons with lower calf survival, worse body conditions and prolonged inter-birth intervals were also confronted with human activities related to pile driving, seismic surveys, platform and pipeline installations and dredging activities. Although impact studies have found changes in behavior, abundance and distribution of gray whales relative to some of these activities within some feeding seasons^40,42,43,46–48^, other studies suffer from limited sample size and inability to address cumulative impacts for the entire foraging season. For example, behavioral and distribution changes during a seismic survey in 2010 found relatively minor changes in gray whale movement, respiration and habitat use relative to sound exposures^42,43,46^. However, these studies had limited sample size due to the timing and duration of the seismic survey and did not account for impacts of a subsequent seismic survey that occurred in the same feeding season that had a longer duration and likely greater exposure to the population. The impacts of these activities cannot be excluded as a potential source of influence relative to calf survival and growth rates of this population.

Compared to their eastern counterparts, the western gray whale population is likely more susceptible to ice conditions due to their spatially static and geographically small foraging grounds. In addition, the western gray whale small population size, high site fidelity and limited observations of the population feeding in alternative areas would make them less resilient to environmental changes^49^. Our results imply that the timing of sea ice cover can predict the post-weaning survival rate, which affects population growth and, thus, recovery of this endangered population of whales. Alternative feeding areas within the Sea of Okhotsk are currently unknown. Given the high site fidelity and residency on the Sakhalin foraging grounds, there might be no other comparable prey resources available in situations when ice or other environmental and/or anthropogenic factors prevent access to their annual foraging areas.

## Methods

### Photo-identification

Individual distinctive features on gray whales’ sides and flukes were photographically captured off northeastern Sakhalin Island, Russia each summer from 2002–2017. Standard photo-identification methods were applied to data collection and processing procedures^50^. Pigmentation patterns on the whales body was the primary feature used to identify individuals, with scars and barnacle patches being secondary identification markings. Annual photo-identification efforts varied in timing (typically July – September), platforms (vessel, drone and/or shore-based) and number of teams. Photo-identification surveys were also conducted annually in both offshore and nearshore feeding areas with varying effort (Fig. 2). Summary of effort and methodological details are documented in Tyurneva *et al*.^34^.

Calf survival was based on the subsequent return and resighting of calves to the feeding grounds in future years. Calves that were not seen in the subsequent four years or beyond of their birth year were presumed to have not survived or permanently emigrated from the area. For example, a calf identified in 2013 but not observed to return to the feeding grounds off Sakhalin by 2017 was presumed to have died or migrated permanently to another foraging area. A minimal of four years of data was chosen to allow photo-identification research effort enough time to recapture the calf in the study area. Photo-identification efforts off Sakhalin have demonstrated considerable high site fidelity and annual return of non-calf individuals to the foraging grounds. However, calves have been observed to have higher variability in their return to the foraging grounds and are generally excluded from population models due to their apparent lower survival (0.68–0.70)^8,10^. For this study, calf survival was estimated based on the percentage of calves observed to have returned to the feeding grounds within the following four years or more compared to the initial number of calves observed during their birth year. For example, if 10 calves were observed in 2010 but only five calves were observed to have returned in 2014–2017 then calf survival would be 50%. Calf survival was also calculated per age with 95% confidence intervals being derived from bootstrapping methods using 1000 iterations of the mean survival.

### Sea-ice concentration

To examine the number of foraging days for a given feeding season, daily ice concentration data, based on passive microwave sensor data from the SSMR and SSMI satellites^51^, were extracted for a feeding season. These remote sensing data were downloaded from the National Snow and Ice Data Center (NSIDC; http://nsidc.org). The gridded resolution of each raster file was 25 km^2^. The median value of grids overlapping the historical known western gray whale nearshore feeding grounds^52^ was calculated on a daily basis for the period of 15 May–15 December in 2002–2017 (Figs. 2, 3). Median ice concentration values of 40% or lower were assumed to be adequate for gray whale foraging within their feeding habitat while higher values were assumed to limit access to their feeding grounds. Sensitivity analyses were also conducted to examine the definition of the foraging season based on alternative ice concentration thresholds. The total number of foraging days was summed based on the number of median daily concentration values ≤ 40% ice concentration values. All sea ice concentration data were processed with R using the raster package^53,54^.

## Data Availability

National Snow and Ice Data Center (NSIDC; http://nsidc.org) provides public access to sea ice concentration data. Photo-identification reports (http://iucn.org/sites/dev/files/content/documents/wgwap_18-19-en_18_2016_jp_photoid_report_final_en.pdf) to the Western Gray Whale Advisory Panel provides detailed data relative to calf production, return and survival.
